# Random forest versus logistic regression: a large-scale benchmark experiment

**DOI:** 10.1186/s12859-018-2264-5

**Published:** 2018-07-17

**Authors:** Raphael Couronné, Philipp Probst, Anne-Laure Boulesteix

**Affiliations:** Department of Medical Information Processing, Biometry and Epidemiology, LMU Munich, Marchioninistr. 15, Munich, 81377 Germany

**Keywords:** Logistic regression, Classification, Prediction, Comparison study

## Abstract

**Background and goal:**

The Random Forest (RF) algorithm for regression and classification has considerably gained popularity since its introduction in 2001. Meanwhile, it has grown to a standard classification approach competing with logistic regression in many innovation-friendly scientific fields.

**Results:**

In this context, we present a large scale benchmarking experiment based on 243 real datasets comparing the prediction performance of the original version of RF with default parameters and LR as binary classification tools. Most importantly, the design of our benchmark experiment is inspired from clinical trial methodology, thus avoiding common pitfalls and major sources of biases.

**Conclusion:**

RF performed better than LR according to the considered accuracy measured in approximately 69% of the datasets. The mean difference between RF and LR was 0.029 (95%-CI =[0.022,0.038]) for the accuracy, 0.041 (95%-CI =[0.031,0.053]) for the Area Under the Curve, and − 0.027 (95%-CI =[−0.034,−0.021]) for the Brier score, all measures thus suggesting a significantly better performance of RF. As a side-result of our benchmarking experiment, we observed that the results were noticeably dependent on the inclusion criteria used to select the example datasets, thus emphasizing the importance of clear statements regarding this dataset selection process. We also stress that neutral studies similar to ours, based on a high number of datasets and carefully designed, will be necessary in the future to evaluate further variants, implementations or parameters of random forests which may yield improved accuracy compared to the original version with default values.

**Electronic supplementary material:**

The online version of this article (10.1186/s12859-018-2264-5) contains supplementary material, which is available to authorized users.

## Introduction

In the context of low-dimensional data (i.e. when the number of covariates is small compared to the sample size), logistic regression is considered a standard approach for binary classification. This is especially true in scientific fields such as medicine or psycho-social sciences where the focus is not only on prediction but also on explanation; see Shmueli [1] for a discussion of this distinction. Since its invention 17 years ago, the random forest (RF) prediction algorithm [2], which focuses on prediction rather than explanation, has strongly gained popularity and is increasingly becoming a common “standard tool” also used by scientists without any strong background in statistics or machine learning. Our experience as authors, reviewers and readers is that random forest can now be used routinely in many scientific fields without particular justification and without the audience strongly questioning this choice. While its use was in the early years limited to innovation-friendly scientists interested (or experts) in machine learning, random forests are now more and more well-known in various non-computational communities.

In this context, we believe that the performance of RF should be systematically investigated in a large-scale benchmarking experiment and compared to the current standard: logistic regression (LR). We make the—admittedly somewhat controversial—choice to consider the standard version of RF only with default parameters — as implemented in the widely used R package randomForest [3] version 4.6-12 — and logistic regression only as the standard approach which is very often used for low dimensional binary classification.

The rationale behind this simplifying choice is that, to become a “standard method” that users with different (possibly non-computational) backgrounds select by default, a method should be simple to use and not require any complex human intervention (such as parameter tuning) demanding particular expertise. Our experience from statistical consulting is that applied research practitioners tend to apply methods in their simplest form for different reasons including lack of time, lack of expertise and the (critical) requirement of many applied journals to keep data analysis as simple as possible. Currently, the simplest approach consists of running RF with default parameter values, since no unified and easy-to-use tuning approach has yet established itself. It is not the goal of this paper to discuss how to improve RF’s performance by appropriate tuning strategies and which level of expertise is ideally required to use RF. We simply acknowledge that the standard variant with default values is widely used and conjecture that things will probably not dramatically change in the short term. That is why we made the choice to consider RF with default values as implemented in the very widely used package randomForest—while admitting that, if time and competence are available, more sophisticated strategies may often be preferable. As an outlook, we also consider RF with parameters tuned using the recent package tuneRanger [4] in a small additional study.

Comparison studies published in literature often include a large number of methods but a relatively small number of datasets [5], yielding an ill-posed problem as far as statistical interpretation of benchmarking results are concerned. In the present paper we take an opposite approach: we focus on only two methods for the reasons outlined above but design our benchmarking experiments in such a way that it yields solid evidence. A particular strength of our study is that we as authors are equally familiar with both methods. Moreover, we are “neutral” in the sense that we have no personal *priori* preference for one of the methods: ALB published a number of papers on RF, but also papers on regression-based approaches [6, 7] and papers pointing to critical problems of RF [8–10]. Neutrality and equal expertise would be much more difficult if not impossible to ensure if several variants of RF (including tuning strategies) and logistic regression were included in the study. Further discussions of the concept of authors’ neutrality can be found elsewhere [5, 11].

Most importantly, the design of our benchmark experiment is inspired by the methodology of clinical trials that has been developed with huge efforts for several decades. We follow the line taken in our recent paper [11] and carefully define the design of our benchmark experiments including, beyond issues related to neutrality outlined above, considerations on sample size (i.e. number of datasets included in the experiment) and inclusion criteria for datasets. Moreover, as an analogue to subgroup analyses and the search for biomarkers of treatment effect in clinical trials, we also investigate the dependence of our conclusions on datasets’ characteristics.

As an important by-product of our study, we provide empirical insights into the importance of inclusion criteria for datasets in benchmarking experiments and general critical discussions on design issues and scientific practice in this context. The goal of our paper is thus two-fold. Firstly we aim to present solid evidence on the performance of standard logistic regression and random forests with default values. Secondly, we demonstrate the design of a benchmark experiment inspired from clinical trial methodology.

The rest of this paper is structured as follows. After a short overview of LR and RF, the associated VIM, partial dependence plots [12], the cross-validation procedure and performance measures used to evaluate the methods (“Background” section), we present our benchmarking approach in “Methods” section, including the criteria for dataset selection. Results are presented in “Results” section.

## Background

This section gives a short overview of the (existing) methods involved in our benchmarking experiments: logistic regression (LR), random forest (RF) including variable importance measures, partial dependence plots, and performance evaluation by cross-validation using different performance measures.

### Logistic regression (LR)

Let *Y* denote the binary response variable of interest and *X*_1_,…,*X*_*p*_ the random variables considered as explaining variables, termed *features* in this paper. The logistic regression model links the conditional probability *P*(*Y*=1|*X*_1_,...,*X*_*p*_) to *X*_1_,…,*X*_*p*_ through

1 $$ P(Y=1|X_{1},...,X_{p})=\frac{\exp\left(\beta_{0}+\beta_{1}X_{1}+\dots+\beta_{p}X_{p}\right)}{1+\exp\left(\beta_{0}+\beta_{1}X_{1}+\dots+\beta_{p}X_{p}\right)},  $$

where *β*_0_,*β*_1_,…,*β*_*p*_ are regression coefficients, which are estimated by maximum-likelihood from the considered dataset. The probability that *Y*=1 for a new instance is then estimated by replacing the *β*’s by their estimated counterparts and the *X*’s by their realizations for the considered new instance in Eq. (1). The new instance is then assigned to class *Y*=1 if *P*(*Y*=1)>*c*, where *c* is a fixed threshold, and to class *Y*=0 otherwise. The commonly used threshold *c*=0.5, which is also used in our study, yields a so-called Bayes classifier. As for all model-based methods, the prediction performance of LR depends on whether the data follow the assumed model. In contrast, the RF method presented in the next section does not rely on any model.

### Random forest (RF)

#### Brief overview

The random forest (RF) is an “ensemble learning” technique consisting of the aggregation of a large number of decision trees, resulting in a reduction of variance compared to the single decision trees. In this paper we consider Leo Breiman’s original version of RF [2], while acknowledging that other variants exist, for example RF based on conditional inference trees [13] which address the problem of variable selection bias [14] and perform better in some cases, or extremely randomized trees [15].

In the original version of RF [2], each tree of the RF is built based on a bootstrap sample drawn randomly from the original dataset using the CART method and the Decrease Gini Impuritiy (DGI) as the splitting criterion [2]. When building each tree, at each split, only a given number mtry of randomly selected features are considered as candidates for splitting. RF is usually considered a black-box algorithm, as gaining insight on a RF prediction rule is hard due to the large number of trees. One of the most common approaches to extract from the random forest interpretable information on the contribution of different variables consists in the computation of the so-called variable importance measures outlined in “Variable importance measures” section. In this study we use the package randomForest [3] (version 4.6-12) with default values, see the next paragraph for more details on tuning parameters.

#### Hyperparameters

This section presents the most important parameters for RF and their common default values as implemented in the R package randomForest [3] and considered in our study. Note, however, that alternative choices may yield better performance [16, 17] and that parameter tuning for RF has to be further addressed in future research. The parameter ntree denotes the number of trees in the forest. Strictly speaking, ntree is not a tuning parameter (see [18] for more insight into this issue) and should be in principle as large as possible so that each candidate feature has enough opportunities to be selected. In practice, however, performance reaches a plateau with a few hundreds of trees for most datasets [18]. The default value is ntree =500 in the package randomForest. The parameter mtry denotes the number of features randomly selected as candidate features at each split. A low value increases the chance of selection of features with small effects, which may contribute to improved prediction performance in cases where they would otherwise be masked by features with large effects. A high value of mtry reduces the risk of having only non-informative candidate features. In the package randomForest, the default value is $\sqrt {p}$ for classification with *p* the number of features of the dataset. The parameter nodesize represents the minimum size of terminal nodes. Setting this number larger yields smaller trees. The default value is 1 for classification. The parameter replace refers to the resampling scheme used to randomly draw from the original dataset different samples on which the trees are grown. The default is replace =TRUE, yielding bootstrap samples, as opposed to replace =FALSE yielding subsamples— whose size is determined by the parameter sampsize.

The performance of RF is known to be relatively robust against parameter specifications: performance generally depends less on parameter values than for other machine learning algorithms [19]. However, noticeable improvements may be achieved in some cases [20]. The recent R package tuneRanger [4] allows to automatically tune RF’s parameters simultaneously using an efficient model-based optimization procedure. In additional analyses presented in “Additional analysis: tuned RF” section, we compare the performance of RF and LR with the performance of RF tuned with this procedure (denoted as TRF).

#### Variable importance measures

As a byproduct of random forests, the built-in variable importance measures (VIM) rank the *variables* (i.e. the features) with respect to their relevance for prediction [2]. The so-called Gini VIM has shown to be strongly biased [14]. The second common VIM, called permutation-based VIM, is directly based on the accuracy of RF: it is computed as the mean difference (over the ntree trees) between the OOB errors before and after randomly permuting the values of the considered variable. The underlying idea is that the permutation of an important feature is expected to decrease accuracy more strongly than the permutation of an unimportant variable.

VIMs are not sufficient in capturing the patterns of dependency between features and response. They only reflect—in the form of a single number—the strength of this dependency. Partial dependence plots can be used to address this shortcoming. They can essentially be applied to any prediction method but are particularly useful for black-box methods which (in contrast to, say, generalized linear models) yield less interpretable results.

### Partial dependence plots

**Fig. 1:**
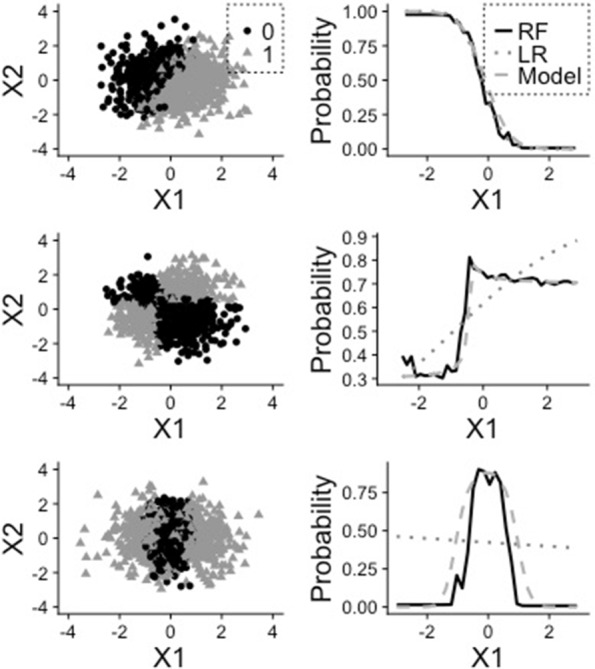
Example of partial dependence plots. Plot of the PDP for the three simulated datasets. Each line is related to a dataset. On the left, visualization of the dataset. On the right, the partial dependence for the variable *X*_1_. First dataset: *β*_0_=1,*β*_1_=5,*β*_2_=−2 (linear), second dataset: *β*_0_=1,*β*_1_=1,*β*_2_=−1,*β*_3_=3 (interaction), third dataset *β*_0_=−2,*β*_4_=5 (non-linear)

Partial dependence plots (PDPs) offer insight of any black box machine learning model, visualizing how each feature influences the prediction while averaging with respect to all the other features. The PDP method was first developed for gradient boosting [12]. Let *F* denote the function associated with the classification rule: for classification, *F*(*X*_1_,…,*X*_*p*_)∈[0,1] is the predicted probability of the observation belonging to class 1. Let *j* be the index of the chosen feature *X*_*j*_ and $X_{\overline {j}}$ its complement, such that $X_{\overline {j}} = \left \{X_{1},...,X_{j-1},X_{j+1},...,X_{p}\right \}$. The partial dependence of *F* on feature *X*_*j*_ is the expectation

2 $$ F_{X_{j}} = \mathbb{E}_{X_{\overline{j}}}F\left(X_{j},X_{\overline{j}}\right)  $$

which can be estimated from the data using the empirical distribution

3 $$ \hat{p}_{X_{j}}(x) = \frac{1}{N} \sum_{i=1}^{N} F\left(x_{i,1},...x_{i,j-1},x,x_{i,j+1},...,x_{i,p}\right),  $$

where *x*_*i*,1_,…,*x*_*i*,*p*_ stand for the observed values of *X*_1_,…,*X*_*p*_ for the *i*th observation. As an illustration, we display in Fig. 1 the partial dependence plots obtained by logistic regression and random forest for three simulated datasets representing classification problems, each including *n*=1000 independent observations. For each dataset the variable *Y* is simulated according to the formula $\log (P(Y=1)/P(Y=0))=\beta _{0}+\beta _{1}X_{1}+\beta _{2}X_{2}+\beta _{3}X_{1}X_{2}+\beta _{4}X_{1}^{2}$. The first dataset (top) represents the linear scenario (*β*_1_≠0, *β*_2_≠0, *β*_3_=*β*_4_=0), the second dataset (middle) an interaction (*β*_1_≠0, *β*_2_≠0, *β*_3_≠0, *β*_4_=0) and the third (bottom) a case of non-linearity (*β*_1_=*β*_2_=*β*_3_=0, *β*_4_≠0). For all three datasets the random vector (*X*_1_,*X*_2_)^⊤^ follows distribution $\mathcal {N}_{2}(0,I)$, with *I* representing the identity matrix. The data points are represented in the left column, while the PDPs are displayed in the right column for RF, logistic regression as well as the true logistic regression model (i.e. with the true coefficient values instead of fitted values). We see that RF captures the dependence and non-linearity structures in cases 2 and 3, while logistic regression, as expected, is not able to.

### Performance assessment

#### Cross-validation

In a *k*-fold cross-validation (CV), the original dataset is randomly partitioned into *k* subsets of approximately equal sizes. At each of the *k* CV iterations, one of the folds is chosen as the test set, while the *k*−1 others are used for training. The considered performance measure is computed based on the test set. After the *k* iterations, the performances are finally averaged over the iterations. In our study, we perform 10 repetitions of stratified 5-fold CV, as commonly recommended [21]. In the stratified version of the CV, the folds are chosen such that the class frequencies are approximately the same in all folds. The stratified version is chosen mainly to avoid problems with strongly imbalanced datasets occurring when all observations of a rare class are included in the same fold. By “10 repetitions”, we mean that the whole CV procedure is repeated for 10 random partitions into *k* folds with the aim to provide more stable estimates.

In our study, this procedure is applied to different performance measures outlined in the next subsection, for LR and RF successively and for *M* real datasets successively. For each performance measure, the results are stored in form of an *M*×2 matrix.

#### Performance measures

Given a classifier and a test dataset of size *n*_*test*_, let $\hat {p}_{i}$, *i*=1,…,*n* denote the estimated probability of the *i*th observation (*i*=1,…,*n*_*test*_) to belong to class *Y*=1, while the true class membership of observation *i* is simply denoted as *y*_*i*_. Following the Bayes rule implicitly adopted in LR and RF, the predicted class $\hat {y}_{i}$ is simply defined as $\hat {y}_{i}=1$ if $\hat {p}_{i}>0.5$ and 0 otherwise.

The *accuracy*, or proportion of correct predictions is estimated as

$$acc = \frac{1}{n_{{test}}}\sum\limits_{i=1}^{n_{t}est}I\left(y_{i}=\hat{y}_{i}\right), $$

where *I*(.) denotes the indicator function (*I*(*A*)=1 if *A* holds, *I*(*A*)=0 otherwise). The *Area Under Curve* (AUC), or probability that the classifier ranks a randomly chosen observation with *Y*=1 higher than a randomly chosen observation with *Y*=0 is estimated as

$$auc = \frac{1}{n_{0,test}n_{1,test}}\sum_{i:y_{i}=1}\sum_{j:y_{j}=0} I\left(\hat{p}_{i}>\hat{p}_{j}\right), $$

where *n*_0,*test*_ and *n*_1,*test*_ are the numbers of observations in the test set with *y*_*i*_=0 and *y*_*i*_=1, respectively. The *Brier score* is a commonly and increasingly used performance measure [22, 23]. It measures the deviation between true class and predicted probability and is estimated as

$$brier = \frac{1}{n_{{test}}}\sum_{i=1}^{n_{{test}}} \left(\hat{p}_{i}-y_{i}\right)^{2}. $$

## Methods

### The OpenML database

So far we have stated that the benchmarking experiment uses a collection of *M* real datasets without further specifications. In practice, one often uses already formatted datasets from public databases. Some of these databases offer a user-friendly interface and good documentation which facilitate to some extent the preliminary steps of the benchmarking experiment (search for datasets, data download, preprocessing). One of the most well-known database is the UCI repository [24]. Specific scientific areas may have their own databases, such as ArrayExpress for molecular data from high-throughput experiments [25]. More recently, the OpenML database [26] has been initiated as an exchange platform allowing machine learning scientists to share their data and results. This database included as many as 19660 datasets in October 2016 when we selected datasets to initiate our study, a non-negligible proportion of which are relevant as example datasets for benchmarking classification methods.

### Inclusion criteria and subgroup analyses

When using a huge database of datasets, it becomes obvious that one has to define criteria for inclusion in the benchmarking experiment. Inclusion criteria in this context do not have any long tradition in computational science. The criteria used by researchers—including ourselves before the present study—to select datasets are most often completely non-transparent. It is often the fact that they select a number of datasets which were found to somehow fit the scope of the investigated methods, but without clear definition of this scope.

We conjecture that, from published studies, datasets are occasionally removed from the experiment *a posteriori* because the results do not meet the expectations/hopes of the researchers. While the vast majority of researchers certainly do not cheat consciously, such practices may substantially introduce bias to the conclusion of a benchmarking experiment; see previous literature [27] for theoretical and empirical investigation of this problem. Therefore, “fishing for datasets” after completion of the benchmark experiment should be prohibited, see Rule 4 of the “ten simple rules for reducing over-optimistic reporting” [28].

Independent of the problem of fishing for significance, it is important that the criteria for inclusion in the benchmarking experiment are clearly stated as recently discussed [11]. In our study, we consider simple datasets’ characteristics, also termed “meta-features”. They are presented in Table 1. Based on these datasets’ characteristics, we define subgroups and repeat the benchmark study within these subgroups, following the principle of subgroup analyses in clinical research. For example, one could analyse the results for “large” datasets (*n*>1000) and “small datasets” (*n*≤1000) separately. Moreover, we also examine the subgroup of datasets related to biosciences/medicine.

**Table 1:** Considered meta-features

Meta-feature	Description
*n*	Number of observations
*p*	Number of features
$\frac {p}{n}$	Dimensionality
*d*	Number of features of the associated design matrix for LR
$\frac {d}{n}$	Dimensionality of the design matrix
*p*_*numeric*_	Number of numeric features
*p*_*categorical*_	Number of categorical features
*p*_*numeric,rate*_	Proportion of numeric features
*C*_*max*_	Percentage of observation of the majority class
*time*	Duration for the run a 5-fold CV with a default Random Forest

### Meta-learning

Taking another perspective on the problem of benchmarking results being dependent on dataset’s meta-features, we also consider modelling the difference between the methods’ performances (considered as response variable) based on the datasets’ meta-features (considered as features). Such a modelling approach can be seen as a simple form of *meta-learning*—a well-known task in machine learning [29]. A similar approach using linear mixed models has been recently applied to the selection of an appropriate classification method in the context of high-dimensional gene expression data analysis [30]. Considering the potentially complex dependency patterns between response and features, we use RF as a prediction tool for this purpose.

### Power calculation

Considering the *M*×2 matrix, collecting the performance measures for the two investigated methods (LR and RF) on the *M* considered datasets, one can perform a test for paired samples to compare the performances of the two methods [31]. We refer to the previously published statistical framework [31] for a precise mathematical definition of the tested null-hypothesis in the case of the t-test for paired samples. In this framework, the datasets play the role of the *i.i.d.* observations used for the t-test. Sample size calculations for the t-test for paired samples can give an indication of the rough number of datasets required to detect a given difference *δ* in performances considered as relevant for a given significance level (e.g., *α*=0.05) and a given power (e.g., 1−*β*=0.8). For large numbers and a two-sided test, the required number of datasets can be approximated as

4 $$ M_{{req}}\approx \frac{\left(z_{1-\alpha/2}+z_{1-\beta}\right)^{2}\sigma^{2}}{\delta^{2}}  $$

where *z*_*q*_ is the *q*-quantile of the normal distribution and *σ*^2^ is the variance of the difference between the two methods’ performances over the datasets, which may be roughly estimated through a pilot study or previous literature.

For example, the required number of datasets to detect a difference in performances of *δ*=0.05 with *α*=0.05 and 1−*β*=0.8 is *M*_*req*_=32 if we assume a variance of *σ*^2^=0.01 and *M*_*req*_=8 for *σ*^2^=0.0025. It increases to *M*_*req*_=197 and *M*_*req*_=50, respectively, for differences of *δ*=0.02.

### Availability of data and materials

Several R packages are used to implement the benchmarking study: mlr (version 2.10) for higher abstraction and a simpler way to conduct benchmark studies [32], OpenML (version 1.2) for loading the datasets [33], and batchtools (version 0.9.2) for parallel computing [34]. Note that the LR and RF learners called via mlr are wrappers on the functions glm and randomForest, respectively.

The datasets supporting the conclusions of this article are freely available in OpenML as described in “The OpenML database” section.

Emphasis is placed on the reproducibility of our results. Firstly, the code implementing all our analyses is fully available from GitHub [35]. For visualization-only purposes, the benchmarking results are available from this link, so that our graphics can be quickly generated by mouse-click. However, the code to re-compute these results, i.e. to conduct the benchmarking study, is also available from GitHub. Secondly, since we use specific versions of R and add-on packages and our results may thus be difficult to reproduce in the future due to software updates, we also provide a docker image [36]. Docker automates the deployment of applications inside a so called “Docker container” [37]. We use it to create an R environment with all the packages we need in their correct version. Note that docker is not necessary here (since all our codes are available from GitHub), but very practical for a reproducible environment and thus for reproducible research in the long term.

## Results

In our study we consider a set of *M* datasets (see “Included datasets” section for more details) and compute for each of them the performance of random forest and logistic regression according to the three performance measures outlined in “Performance assessment” section.

### Included datasets

**Fig. 2:**
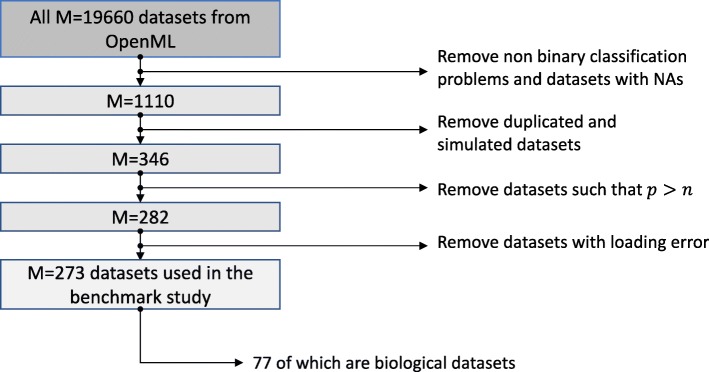
Selection of datasets. Flowchart representing the criteria for selection of the datasets

From approximately 20000 datasets currently available from OpenML [26], we select those featuring binary classification problems. Further, we remove the datasets that include missing values, the obviously simulated datasets as well as duplicated datasets. We also remove datasets with more features than observations (*p*>*n*), and datasets with loading errors. This leaves us with a total of 273 datasets. See Fig. 2 for an overview.

### Missing values due to errors

Out of the 273 selected datasets, 8 require too much computing time when parallelized using the package batchtools and expired or failed. These—extremely large—datasets are discarded in the rest of the study, leaving us with 265 datasets.

Both LR and RF fail in the presence of categorical features with too many categories. More precisely, RF fails when more than 53 categories are detected in at least one of the features, while LR fails when levels undetected during the training phase occur in the test data. We could admittedly have prevented these errors through basic preprocessing of the data such as the removal or recoding of the features that induce errors. However, we decide to just remove the datasets resulting in NAs because we do not want to address preprocessing steps, which would be a topic on their own and cannot be adequately treated along the way for such a high number of datasets. Since 22 datasets yield NAs, our study finally includes 265-22 =243 datasets.

### Main results

**Fig. 3:**
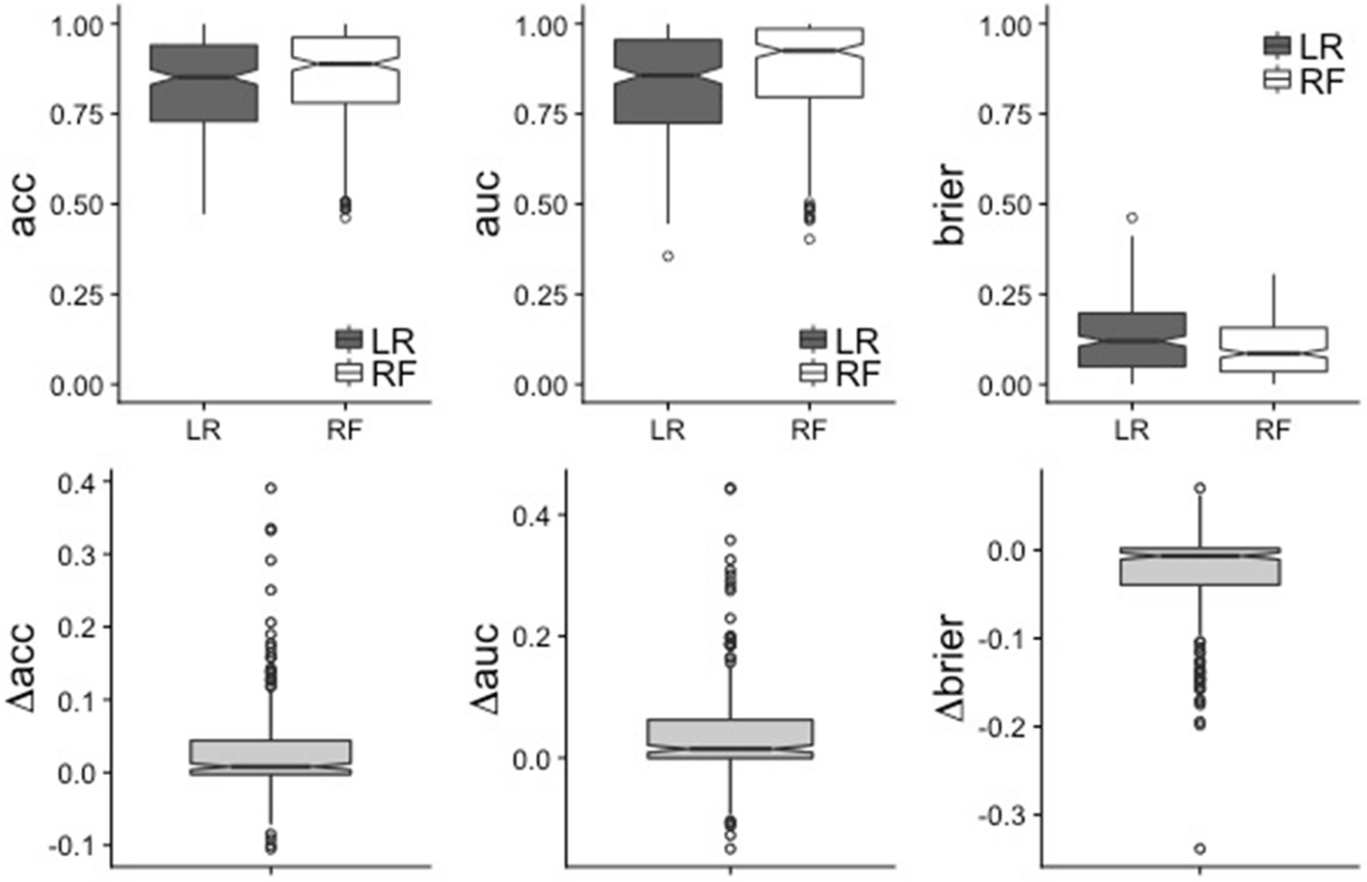
Main results of the benchmark experiment. Boxplots of the performance for the three considered measures on the 243 considered datasets. Top: boxplot of the performance of LR (dark) and RF (white) for each performance measure. Bottom: boxplot of the difference of performances *Δperf*=*perf*_*RF*_−*perf*_*LR*_

Overall performances are presented in a synthesized form in Table 2 for all three measures in form of average performances along with standard deviations and confidence intervals computed using the adjusted bootstrap percentile (BCa) method [38]. The boxplots of performances of Random Forest (RF) and Logistic Regression (LR) for the three considered performance measures are depicted in Fig. 3, which also includes the boxplot of the difference in performances (bottom row). It can be seen from Fig. 3 that RF performs better for the majority of datasets (69.0% of the datasets for *acc*, 72.3% for *auc* and 71.5% for *brier*). Furthermore, when LR outperforms RF the difference is small. It can also be noted that the differences in performance tend to be larger for *auc* than for *acc* and *brier*.

**Table 2:** Performances of LR and RF (top: accuracy, middle: AUC, bottom: Brier score): (top: accuracy, middle: AUC, bottom: Brier score): mean performance *μ*, standard deviation *σ* and confidence interval for the mean (estimated via the bootstrap BCa method [38]) on the 243 datasets

Acc	*μ*	*σ*	BCa confidence interval
Logistic regression	0.826	0.135	[0.808, 0.842]
Random forest	0.854	0.134	[0.837, 0.870]
Difference	0.029	0.067	[0.021, 0.038]
Auc			
Logistic regression	0.826	0.149	[0.807, 0.844]
Random forest	0.867	0.147	[0.847, 0.884]
Difference	0.041	0.088	[0.031, 0.054]
Brier			
Logistic regression	0.129	0.091	[0.117, 0.140]
Random forest	0.102	0.080	[0.092, 0.112]
Difference	-0.0269	0.054	[-0.034, -0.021]

### Explaining differences: datasets’ meta-features

In this section, we now perform different types of additional analyses with the aim to investigate the relation between the datasets’ meta-features and the performance difference between LR and RF. In “Preliminary analysis” section, we first consider an example dataset in detail to examine whether changing the sample size *n* and the number *p* of features for this given dataset changes the difference between performances of LR and RF (focusing on a specific dataset, we are sure that confounding is not an issue). In “Subgroup analyses: meta-features” to “Meta-learning” sections, we then assess the association between dataset’s meta-features and performance difference over all datasets included in our study.

#### Preliminary analysis

While it is obvious to any computational scientist that the performance of methods may depend on meta-features, this issue is not easy to investigate in real data settings because i) it requires a large number of datasets—a condition that is often not fulfilled in practice; ii) this problem is enhanced by the correlations between meta-features. In our benchmarking experiment, however, we consider such a huge number of datasets that an investigation of the relationship between methods’ performances and datasets’ characteristic becomes possible to some extent.

**Fig. 4:**
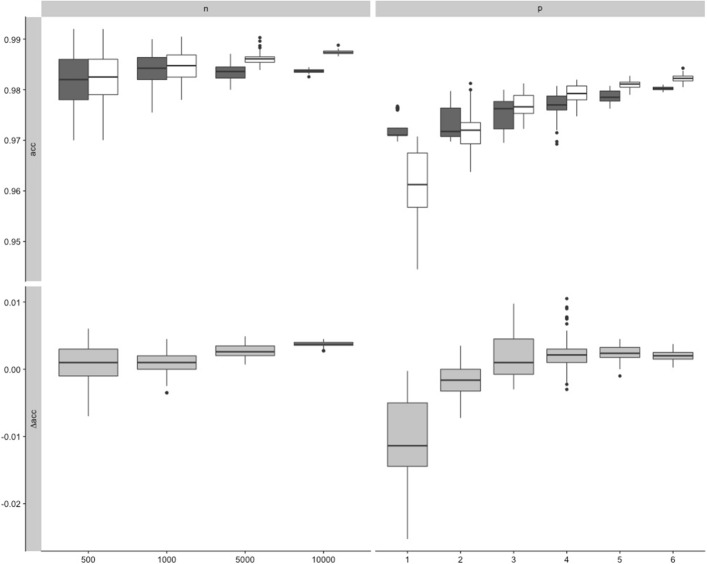
Influence of *n* and *p*: subsampling experiment based on dataset ID=310. Top: Boxplot of the performance (*acc*) of RF (dark) and LR (white) for *N*=50 sub-datasets extracted from the OpenML dataset with ID=310 by randomly picking *n*^′^≤*n* observations and *p*^′^<*p* features. Bottom: Boxplot of the differences in performances *Δacc*=*Acc*_*RF*_−*Acc*_*LR*_ between RF and LR. *p*^′^∈{1,2,3,4,5,6}. *n*^′^∈{5*e*2,1*e*3,5*e*3,1*e*4}. Performance is evaluated through 5-fold-cross-validation repeated 2 times

As a preliminary, let us illustrate this idea using only one (large) biomedical dataset, the OpenML dataset with *ID*=310 including *n*_0_=11183 observations and *p*_0_=7 features. A total of *N*=50 sub-datasets are extracted from this dataset by randomly picking a number *n*^′^<*n*_0_ of observations or a number *p*^′^<*p*_0_ of features. Thereby we successively set *n*^′^ to *n*^′^=5.10^2^,10^3^,5.10^3^,10^4^ and *p*^′^ to *p*^′^=1,2,3,4,5,6. Figure 4 displays the boxplots of the accuracy of RF (white) and LR (dark) for varying *n*^′^ (top-left) and varying *p*^′^ (top-right). Each boxplot represents *N*=50 data points. It can be seen from Fig. 4 that the accuracy increases with *p*^′^ for both LR and RF. This reflects the fact that relevant features may be missing from the considered random subsets of *p*^′^ features. Interestingly, it can also be seen that the increase of accuracy with *p*^′^ is more pronounced for RF than for LR. This supports the commonly formulated assumption that RF copes better with large numbers of features. As a consequence, the difference between RF and LR (bottom-right) increases with *p*^′^ from negative values (LR better than RF) to positive values (RF better than LR). In contrast, as *n* increases the performances of RF and LR increase slightly but quite similarly (yielding a relatively stable difference), while—as expected—their variances decrease; see the left column of Fig. 4.

#### Subgroup analyses: meta-features

To further explore this issue over all 243 investigated datasets, we compute Spearman’s correlation coefficient between the difference in accuracy between random forest and logistic regression (*Δacc*) and various datasets’ meta-features. The results of Spearman’s correlation test are shown in Table 3. These analyses again point to the importance of the number *p* of features (and related meta-features), while the dataset size *n* is not significantly correlated with *Δacc*. The percentage *C*_*max*_ of observations in the majority class, which was identified as influencing the relative performance of RF and LR in a previous study [39] conducted on a dataset from the field of political science is also not significantly correlated with *Δacc* in our study. Note that our results are averaged over a large number of different datasets: they are not incompatible with the existence of an effect in some cases.

**Table 3:** Correlation between *Δacc* and dataset’s features

	Spearman’s *ρ*	Spearman’s *ρp*-value
*n*	-0.0338	6.00·10^−1^
*p*	0.331	1.32·10^−7^
$\frac {p}{n}$	0.254	6.39·10^−5^
*d*	0.258	4.55·10^−5^
$\frac {d}{n}$	0.246	1.04·10^−4^
*p*_*numeric*_	0.254	6.09·10^−5^
*p*_*categorical*_	-0.076	2.37·10^−1^
*p*_*numeric,rate*_	0.240	1.54·10^−4^
*C*_*max*_	0.00735	9.10·10^−1^

To investigate these dependencies more deeply, we examine the performances of RF and LR within subgroups of datasets defined based on datasets’ meta-features (called meta-features from now on), following the principle of subgroup analyses well-known in clinical research. As some of the meta-features displayed in Table 3 are mutually (highly) correlated, we cluster them using a hierarchical clustering algorithm (data not shown). From the resulting dendogram we decide to select the meta-features *p*, *n*, $\frac {p}{n}$, *C*_*max*_, while other meta-features are considered redundant and ignored in further analyses.

**Fig. 5:**
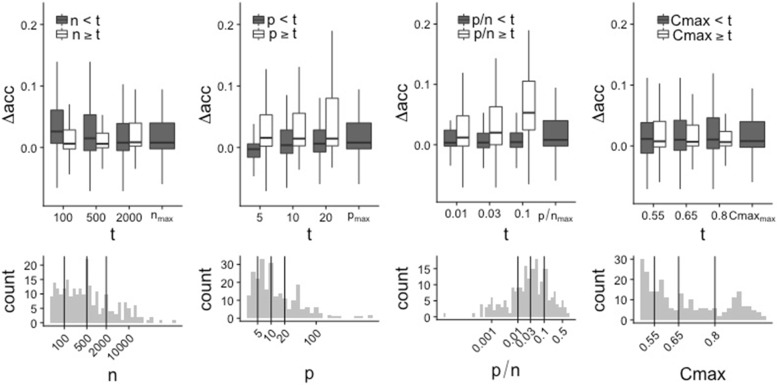
Subgroup analyses. Top: for each of the four selected meta-features *n*, *p*, *p*/*n* and *C*_*max*_, boxplots of *Δacc* for different thresholds as criteria for dataset selection. Bottom: distribution of the four meta-features (log scale), where the chosen thresholds are displayed as vertical lines. Note that outliers are not shown here for a more convenient visualization. For a corresponding figure including the outliers as well as the results for *auc* and *brier*, see Additional file 1

Figure 5 displays the boxplots of the differences in accuracy for different subgroups based on the four selected meta-features *p*, *n*, $\frac {p}{n}$ and *C*_*max*_. For each of the four meta-features, subgroups are defined based on different cut-off values, denoted as *t*, successively. The histograms of the four meta-features for the 243 datasets are depicted in the bottom row of the figure, where the considered cutoff values are materialized as vertical lines. Similar pictures are obtained for the two alternative performance measures *auc* and *brier*; See Additional file 1.

It can be observed from Fig. 5 that RF tends to yield better results than LR for a low *n*, and that the difference decreases with increasing *n*. In contrast, RF performs comparatively poorly for datasets with *p*<5, but better than LR for datasets with *p*≥5. This is due to low performances of RF on a high proportion of the datasets with *p*<5. For $\frac {p}{n}$, the difference between RF and LR is negligible in low dimension $\left (\frac {p}{n}<0.01\right)$, but increases with the dimension. The contrast is particularly striking between the subgroups $\frac {p}{n}<0.1$ (yielding a small *Δacc*) and $\frac {p}{n}\geq 0.1$ (yielding a high *Δacc*), again confirming the hypothesis that the superiority of RF over LR is more pronounced for larger dimensions.

Note, however, that all these results should be interpreted with caution, since confounding may be an issue.

#### Subgroup analyses: substantive context

**Fig. 6:**
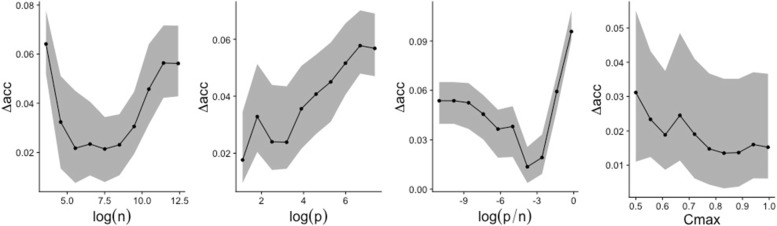
Plot of the partial dependence for the 4 considered meta-features : *log*(*n*), *log*(*p*), $log{\left (\frac {p}{n}\right)}$, *C*_*max*_. The *log* scale was chosen for 3 of the 4 features to obtain more uniform distribution (see Fig. 5 where the distribution is plotted in *log* scale). For each plot, the black line denotes the median of the individual partial dependences, and the lower and upper curves of the grey regions represent respectively the 25%- und 75%-quantiles. Estimated mse is 0.00382 via a 5-CV repeated 4 times

Furthermore, we conduct additional subgroup analyses focusing on the subgroup of datasets from the field of biosciences/medicine. Out of the 243 datasets considered so far, 67 are related to this field. The modified versions of Figs. 3 and 5 and Table 2 (as well as Fig. 6 discussed in “Meta-learning” section) obtained based on the subgroup formed by datasets from biosciences/medicine are displayed in Additional file 2. The outperformance of RF over LR is only slightly lower for datasets from biosciences/medicine than for the other datasets: the difference between datasets from biosciences/medicine and datasets from other fields is not significantly different from 0. Note that one may expect bigger differences between specific subfields of biosciences/medicine (depending on the considered prediction task). Such investigations, however, would require subject matter knowledge on each of these tasks. They could be conducted in future studies by experts of the respective tasks; see also the “Discussion” section.

#### Meta-learning

The previous section showed that benchmarking results in subgroups may be considerably different from that of the entire datasets collection. Going one step further, one can extend the analysis of meta-features towards meta-learning to gain insight on their influence. More precisely, taking the datasets as observations we build a regression RF that predicts the difference in performance between RF and LR based on the four meta-features considered in the previous subsection $\left ({p}, {n}, \frac {p}{n} \text { and } C_{max}\right)$. Figure 6 depicts partial dependence plots for visualization of the influence of each meta-feature. Again, we notice a dependency on *p* and $\frac {p}{n}$ as outlined in “Subgroup analyses: meta-features” section and the comparatively bad results of RF when compared to LR for datasets with small *p*. The importance of *C*_*max*_ and *n* is less noticeable.

Although these results should be considered with caution, since they are possibly highly dependent on the particular distribution of the meta-features over the 243 datasets and confounding may be an issue, we conclude from “Explaining differences: datasets’ meta-features” section that meta-features substantially affect *Δacc*. This points out the importance of the definition of clear inclusion criteria for datasets in a benchmark experiment and of the consideration of the meta-features’ distributions.

### Explaining differences: partial dependence plots

In the previous section we investigated the impact of datasets’ meta-features on the results of benchmarking and modeled the difference between methods’ performance based on these meta-features. In this section, we take a different approach for the explanation of differences. We use partial dependence plots as a technique to assess the dependency pattern between response and features underlying the prediction rule. More precisely, the aim of these additional analyses is to assess whether differences in performances (between LR and RF) are related to differences in partial dependence plots. After getting a global picture for all datasets included in our study, we inspect three interesting “extreme cases” more closely. In a nutshell, we observe no strong correlation between the difference in performances and the difference in partial dependences over the 243 considered datasets. More details are given in Additional file 3: in particular, we see in the third example dataset that, as expected from the theory, RF performs better than LR in the presence of a non-linear dependence pattern between features and response.

### Additional analysis: tuned RF

As an outlook, a third method is compared to RF and LR: RF tuned using the package tuneRanger [4] with all arguments set to the defaults (in particular, tuning is performed by optimizing the Brier score by using the out-of-bag observations). To keep computational time reasonable, in this additional study CV is performed only once (and not repeated 10 times as in the main study), and we focus on the 67 datasets from biosciences/medicine. The results are displayed in Additional file 4 in the same format as the previously described figures.

Tuned RF (TRF) has a slightly better performance than RF: both *acc* and *auc* are on average by 0.01 better for TRF than for RF. Apart from this slight average difference, the performances of RF and TRF appear to be similar with respect to subgroup analyses and partial dependence plots. The most noticeable, but not very surprising result is that improvement through tuning tends to be more pronounced in cases where RF performs poorly (compared to LR).

### Application to C-to-U conversion data

As an illustration, we apply LR, RF and TRF to the C-to-U conversion data previously investigated in relation to random forest in the bioinformatics literature [14, 40]. In summary, RNA editing is the process whereby RNA is modified from the sequence of the corresponding DNA template [40]. For instance, cytidine-to-uridine conversion (abbreviated C-to-U conversion) is common in plant mitochondria. Cummings and Myers [40] suggest to use information from neighboring sequence regions flanking the sites of interest to predict editing status, among others in Arabidopsis thaliana. For each of the 876 complete observations included in the dataset (available at https://static-content.springer.com/esm/art%3A10.1186%2F1471-2105-5-132/MediaObjects/12859_2004_248_MOESM1_ESM.txt), the following features are available:

- the binary response at the site of interest (edited versus not edited)
- the 40 nucleotides at positions -20 to 20, relative to the edited site (4 categories: A, C, T, G), whereby we consider only the nucleotides at positions -5 to 5 as candidates in the present study,
- the codon position *cp* (4 categories: P0, P1, P2, PX),
- the (continuous) estimated folding energy (*fe*)
- the (continuous) difference *dfe* in estimated folding energy between pre-edited and edited sequences.

When evaluating LR and RF on this dataset using the same evaluation procedure as for the OpenML datasets, we see that LR and RF perform very similarly for all three considered measures: 0.722 for LR versus 0.729 for RF for the accuracy (acc), 0.792 for LR versus 0.785 for RF for the Area Under the Curve (auc) and 0.185 for LR versus 0.187 for RF for the Brier score. When looking at permutation variable importances (for RF) and p-values of the Wald test (for LR), we see that the 13 candidate features are assessed similarly by both methods. In particular, the two closest neighbor nucleotides are by far the strongest predictors for both methods.

Using the package ’tuneRanger’ (corresponding to method TRF in our benchmark), the results are extremely similar for all three measures (acc: 0.722, auc: 0.7989, brier: 0.184), indicating that, for this dataset, the default values are adequate. Using the package ’glmnet’ to fit a ridge logistic regression model (with the penalty parameter chosen by internal cross-validation, as done by default in ’glmnet’), the results are also similar: 0.728 for acc, 0.795 for auc and 0.189 for brier.

To gain further insight into the impact of specific tuning parameters, we proceed by running RF with its default parameters except for one parameter, which is set to several candidate values successively. The parameters mtry, nodesize and sampsize are considered successively as varying parameter (while the other two are fixed to the default values). More precisely, mtry is set 1, 3, 5, 10 and 13 successively; nodesize is set to 2, 5, 10, 20 successively; and sampsize is set to 0.5*n* and 0.75*n* successively. The result is that all three performance measures are remarkably robust to changes of the parameters: all accuracy values are between 0.713 and 0.729, all AUC values are between 0.779 and 0.792, and all Brier score values are between 0.183 and 0.197. Large nodesize values seem to perform slightly better (this is in line with the output of tuneRanger, which selects 17 as the optimal nodesize value), while there is no noticeable trend for mtry and sampsize. In conclusion, the analysis of the C-to-U conversion dataset illustrates that one should not expect too much from tuning RF in general (note, however, that tuning may improve performance in other cases, as indicated by our large-scale benchmark study).

## Discussion

### Summary

We presented a large-scale benchmark experiment for comparing the performance of logistic regression and random forest in binary classification settings. The overall results on our collection of 243 datasets showed better accuracy for random forest than for logistic regression for 69.0% of the datasets. On the whole, our results support the increasing use of RF with default parameter values as a standard method—which of course neither means that it performs better on all datasets nor that other parameter values/variants than the default are useless!

We devoted particular attention to the inclusion criteria applied when selecting datasets for our study. We investigated how the conclusions of our benchmark experiment change in different subgroups of datasets. Our analyses reveal a noticeable influence of the number of features *p* and the ratio $\frac {p}{n}$. The superiority of RF tends to be more pronounced for increasing *p* and $\frac {p}{n}$. More generally, our study outlines the importance of inclusion criteria and the necessity to include a large number of datasets in benchmark studies as outlined in previous literature [11, 28, 31].

### Limitations

Firstly, as previously discussed [11], results of benchmarking experiments should be considered as conditional on the set of included datasets. As demonstrated by our analyses on the influence of inclusion criteria for datasets, different sets of datasets yield different results. While the set of datasets considered in our study has the major advantages of being large and including datasets from various scientific fields, it is not strictly speaking representative of a “population of datasets”, hence essentially yielding conditional conclusions.

Secondly, as all real data studies, our study considers datasets following different unknown distributions. It is not possible to control the various datasets’ characteristics that may be relevant with respect to the performance of RF and LR. Simulations fill this gap and often yield some valuable insights into the performance of methods in various settings that a real data study cannot give.

Thirdly, other aspects of classification methods are important but have not been considered in our study, for example issues related to the *transportability* of the constructed prediction rules. By transportability, we mean the possibility for interested researchers to apply a prediction rule presented in the literature to their own data [9, 10]. With respect to transportability, LR is clearly superior to RF, since it is sufficient to know the fitted values of the regression coefficient to apply a LR-based prediction rule. LR also has the major advantage that it yields interpretable prediction rules: it does not only aim at *predicting* but also at *explaining*, an important distinction that is extensively discussed elsewhere [1] and related to the “two cultures” of statistical modelling described by Leo Breiman [41]. These important aspects are not taken into account in our study, which deliberately focuses on prediction accuracy.

Fourthly, our main study was intentionally restricted to RF with default values. The superiority of RF may be more pronounced if used together with an appropriate tuning strategy, as suggested by our additional analyses with TRF. Moreover, the version of RF considered in our study has been shown to be (sometimes strongly) biased in variable selection [14]. More precisely, variables of certain types (e.g., categorical variables with a large number of categories) are systematically preferred by the algorithm for inclusion in the trees irrespectively of their relevance for prediction. Variants of RF addressing this issue [13] may perform better, at least in some cases.

### Outlook

In this paper, we mainly focus on RF with default parameters as implemented in the widely used package randomForest and only briefly consider parameter tuning using a tuning procedure implemented in the package tuneRanger as an outlook. The rationale for this choice was to provide evidence for default values and thereby the analysis strategy most researchers currently apply in practice. The development of reliable and practical parameter tuning strategies, however, is crucial and more attention should be devoted in the future. Tuning strategies should be themselves compared in benchmark studies. Beyond the special case of RF, particular attention should be given to the development of user-friendly tools such as tuneRanger [4], considering that one of the main reasons for using default values is probably the ease-of-use—an important aspect in the hectic academic context. By presenting the results on the average superiority with default values over LR, we by no means want to definitively establish these default values. Instead, our study is intended as a fundamental first step towards well-designed studies providing solid well-delimited evidence on the performance.

Before further studies are performed on tuning strategies, we insist that, whenever performed in applications of RF, parameter tuning should ideally always be reported clearly including all technical details either in the main or in its supplementary materials. Furthermore, the uncertainty regarding the “best tuning strategy” should in no circumstances be exploited for conscious or subconscious “fishing for significance”.

Moreover, our study could also be extended to yield differentiated results for specific prediction tasks, e.g., prediction of disease outcome based on different types of omics data, or prediction of protein structure and function. In the present study, we intentionally considered a broad spectrum of data types to achieve a high number of datasets. Obviously, performance may depend on the particular prediction task, which should be addressed in more focused benchmark studies conducted by experts of the corresponding prediction task with good knowledge of the considered substantive context. However, the more specific the considered prediction task and data type, the more difficult it will be to collect the needed number of datasets to achieve the desired power. In real data studies, there is a trade-off between the homogeneity and the number of available datasets.

## Conclusion

Our systematic large-scale comparison study performed using 243 real datasets on different prediction tasks shows the good *average* prediction performance of random forest (compared to logistic regression) even with the standard implementation and default parameters, which are in some respects suboptimal. This study should in our view be seen both as (i) an illustration of the application of principles borrowed from clinical trial methodology to benchmarking in computational sciences—an approach that could be more widely adopted in this field and (ii) a motivation to pursue research (and comparison studies!) on random forests, not only on possibly better variants and parameter choices but also on strategies to improve their transportability.

## Additional files

Additional file 1 Additional results of subgroup analyses. Additional file 1 extends Fig. 5 for all considered measures, and include the outliers. (PDF 203 kb)

Additional file 2 Datasets from biosciences/medicine. Additional file 2 presents the modified versions of Figs. 3, 5 and 6 as well as Table 2 obtained using the datasets from biosciences/medicine only. (PDF 288 kb)

Additional file 3 Results on partial dependence. Additional file 3 includes a study on interesting extreme cases that allows to gain more insight into the behaviour of LR and RF using partial dependence plots defined in “Partial dependence plots” section. (PDF 256 kb)

Additional file 4 Results with tuned random forest (TRF). Additional file 4 shows the results of the comparison study between LR, RF and TRF based on the 67 datasets from biosciences/medicine. (PDF 224 kb)

## Abbreviations

acc: Accuracy
auc: Area under the curve
brier: Brier score
CV: Cross-validation
LR: Logistic Regression
PDP: Partial dependence plot
RF: Random forest
VIM: Variable importance measure
