# Supplementary material for: Random forest versus logistic regression: a large-scale benchmark experiment
Source: BMC Bioinformatics. 2018 Jul 17;19:270. doi: 10.1186/s12859-018-2264-5 (PMC6050737; doi:10.1186/s12859-018-2264-5)
Supplement: Supplementary file 1 — Additional results of subgroup analyses. Additional file 1 extends Fig. 5 for all considered measures, and include the outliers. (PDF 203 kb) [file 12859_2018_2264_MOESM1_ESM.pdf]

Additional File 1:
Additional results on subgroup analyses

This figure shows the overall results for the inclusion criteria, extending Figure 5 for *auc* and *brier* and (in contrast to Figure 5) also displaying outlying values. We observe that results for different performance measures are close, with similar trends for the boxplots.

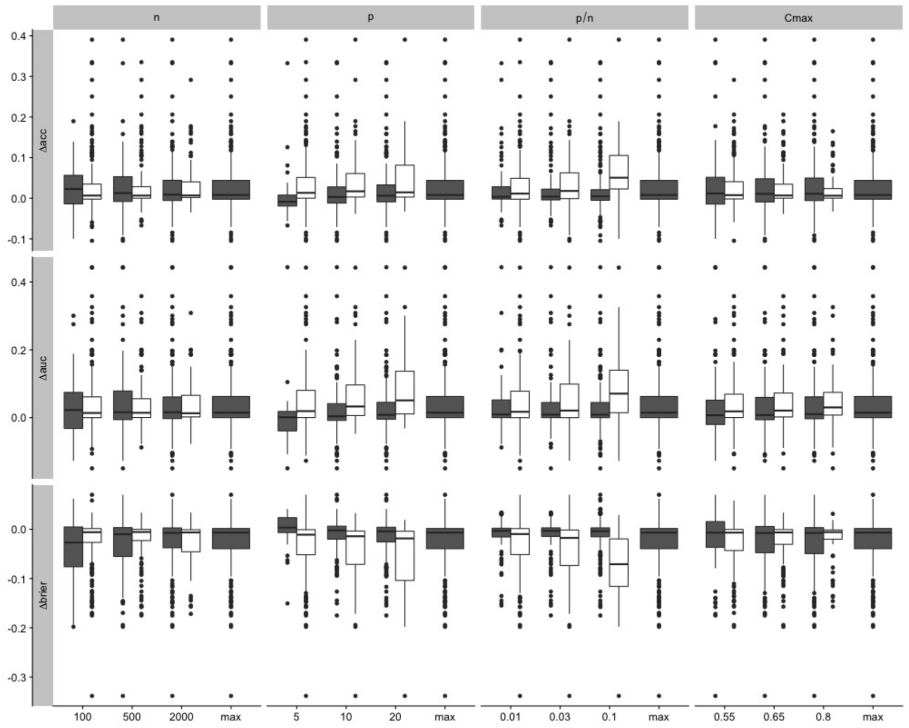

Figure 1 Extension of Figure 5 for all the considered measures. The three rows are respectively dedicated to the three measures acc, auc and $brier$. In each row, we plot four series of boxplots, representing different inclusion criteria for the meta-features n, p, $\frac{p}{n}$ and $Cmax$ as in Figure 5. The dark boxplots represent the observations under the threshold, while the white boxplots represent the observations above the threshold. Note that outliers are shown here.
