# Supplementary material for: Random forest versus logistic regression: a large-scale benchmark experiment
Source: BMC Bioinformatics. 2018 Jul 17;19:270. doi: 10.1186/s12859-018-2264-5 (PMC6050737; doi:10.1186/s12859-018-2264-5)
Supplement: Supplementary file 2 — Datasets from biosciences/medicine. Additional file 2 presents the modified versions of Figs. 3, 5 and 6 as well as Table 2 obtained using the datasets from biosciences/medicine only. (PDF 288 kb) [file 12859_2018_2264_MOESM2_ESM.pdf]

Additional file 2:
Datasets from biosciences/medicine

Main results

We see on Figure 1, which is the equivalent for the 67 datasets from biosciences/medicine of Figure 3 from the paper, that RF performs better than LR for all three measures, but that this superiority is more pronounced for *auc* and *brier*. RF is ranked first for 55 % of the datsets for *acc*, 63 % for *auc* and 63% for *brier*.

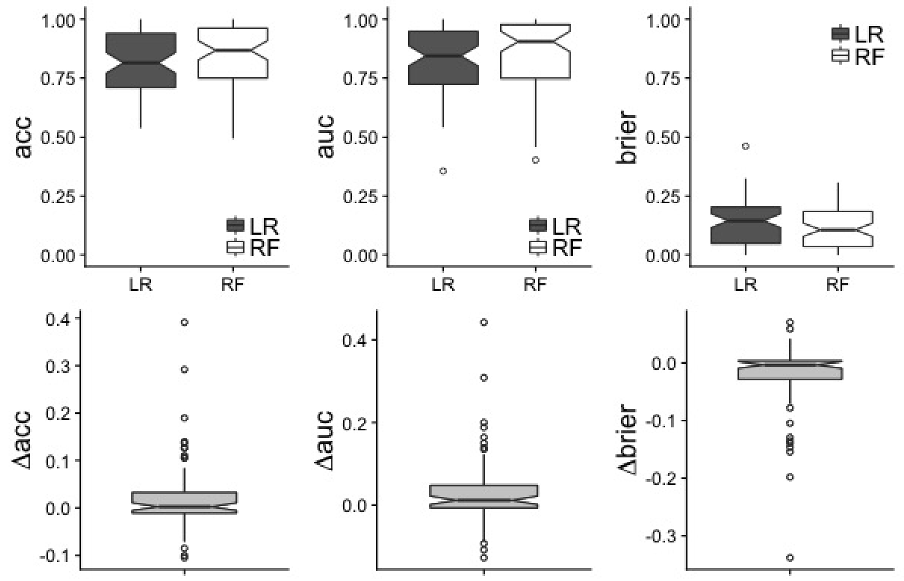

Figure 1 Main results of the benchmark experiment (for the 67 datasets from biosciences/medicine) Boxplots of the performance for the three considered measures on the 67 considered datasets. Top: boxplot of the performance of LR (dark) and RF (white) for each performance measure. Bottom: boxplot of the difference of performances $\Delta perf = perf_{RF} - perf_{LR}$.

Similarly to Table 2 from the paper, Table 1 shows the mean, standard deviation and bootstrap BCa confidence interval of the mean for the performances (according to the three measures *acc*, *auc* and *brier*) of RF and LR and their difference, but for the 67 datasets from biosciences/medicine.

Table 1 Performances of LR and RF (for the 67 datasets from biosciences/medicine) (top: accuracy, middle: AUC, bottom: Brier score): mean performance μ, standard deviation σ and confidence interval for the mean (estimated via the bootstrap BCa method). It can be seen from this table that RF performs significantly better than LR for all three measures.

**acc**	μ	σ	BCa confidence interval
Logistic regression	0.813	0.136	[0.779, 0.842]
Random forest	0.837	0.138	[0.802, 0.868]
Difference	0.024	0.0080	[0.008, 0.048]
**auc**			
Logistic regression	0.818	0.149	[0.779, 0.851]
Random forest	0.850	0.156	[0.809, 0.886]
Difference	0.032	0.089	[0.014, 0.061]
**brier**			
Logistic regression	0.139	0.100	[0.117, 0.163]
Random forest	0.114	0.086	[0.094, 0.136]
Difference	-0.0245	0.064	[-0.043, -0.0012]

Subgroup analyses: meta-features

Figure 2 is the equivalent—for the 67 datasets from biosciences/medicine—of Figure 5 from the paper: it displays the differences between the accuracies of RF and LR in different subgroup of datasets. We observe no noticeable differences between the results for these 67 datasets and the results for all datasets.

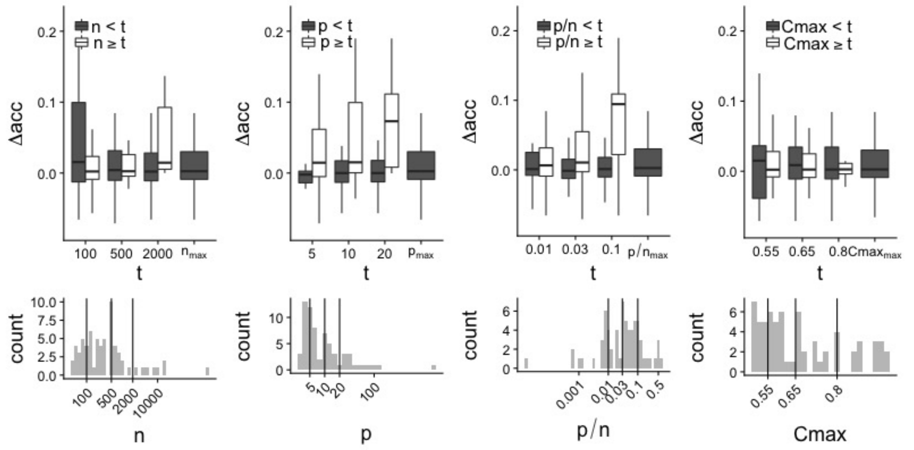

Figure 2 Subgroup analyses (for the 67 datasets from biosciences/medicine) Top: for each of the four selected meta-features n, p, p/n and C_{max}, boxplots of Δacc for different thresholds as criteria for dataset selection. Bottom: distribution of the four meta-features (log scale), where the chosen thresholds are displayed as vertical lines. Note that outliers are not shown here for a more convenient visualization.

Meta-learning

Figure 3 is the equivalent—for the 67 datasets from biosciences/medicine—of Figure 6 from the paper. Again, we do not observe any noticeable difference between the two figures.

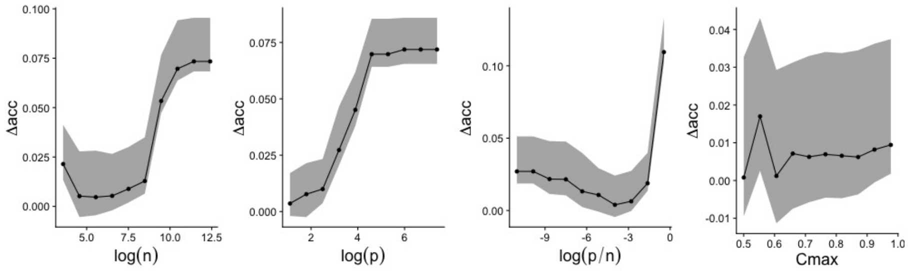

Figure 3 Partial dependence plots (for the 67 datasets from biosciences/medicine) Plot of the partial dependence for the 4 considered meta-features : $log_(n)$, $log_(p)$, $log_(\frac{p}{n})$, C_{max}. The log scale was chosen for 3 of the 4 features to obtain a more uniform distribution. For each plot, the black line denotes the median of the individual partial dependances, and the lower and upper curves of the grey regions represent respectively the 25%- and 75%-quantiles. Estimated mse is 0.0051 via a 5-CV repeated 4 times.

Comparison biosciences/medicine versus other fields

We finally compare the results obtained for the 67 datasets from biosciences/medicine and the results obtained for datasets from other fields. More precisely, Figure 4 displays the boxplots of the differences Δacc, Δauc and $\Delta brier$ between RF and LR for datasets from other fields (grey) and datasets from biosciences/medicine (white). These boxplots are those displayed in the bottom row of Figure 3 from the paper and Figure 1 from this additional file.

It can be seen from Figure 4 that the superiority of RF over LR is slightly more pronounced for datasets from other fields than for datasets from biosciences/medicine (the white boxplots are closer to 0 than the grey boxplots). However, the difference between datasets from biosciences/medicine and datasets from other fields is not significantly different from 0 according to the two-sample t-test (for all three measures acc, auc and $brier$).

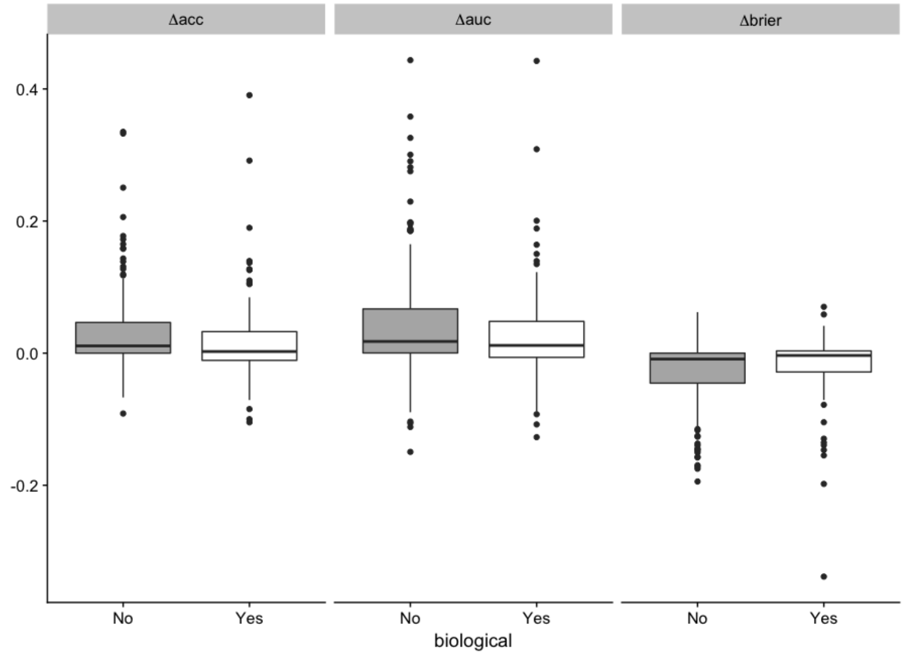

Figure 4 Datasets from biosciences/medicine versus datasets from other fields Boxplots of the difference of performances $\Delta perf = perf_{RF} - perf_{LR}$ for datasets from other fields (grey) and datasets from biosciences/medicine (white).
