# Supplementary material for: Random forest versus logistic regression: a large-scale benchmark experiment
Source: BMC Bioinformatics. 2018 Jul 17;19:270. doi: 10.1186/s12859-018-2264-5 (PMC6050737; doi:10.1186/s12859-018-2264-5)
Supplement: Supplementary file 3 — Results on partial dependence. Additional file 3 includes a study on interesting extreme cases that allows to gain more insight into the behaviour of LR and RF using partial dependence plots defined in “Partial dependence plots” section. (PDF 256 kb) [file 12859_2018_2264_MOESM3_ESM.pdf]

Additional file 3: Results on partial dependence

We further investigate the behavior of logistic regression (LR) and random forest (RF) based on a few interesting example datasets from OpenML by considering partial dependence plots—as we did in subsection 2.3 for simulated datasets. More precisely, the aim of these additional analyses is to assess whether differences in performances (between LR and RF) are related to differences in partial dependence plots. After getting a global picture for all datasets included in our study, we inspect three interesting "extreme cases" more closely. For this purpose we need a measure to quantify the difference between partial dependence plots of two methods (here, LR and RF). Since we did not find such a measure in the literature, we suggest a simple approach in the next section.

Measuring differences in partial dependences

For feature X_j ($j \in 1,..,p$), let $u_{i,j}, i \in 1,..,10$ denote the uniform grid on which the partial dependence is computed, with $u_{1,j} = min(X_j)$ and $u_{10,j} = max(X_j)$. Let $PD_{i,j}^{(RF)}$ and $PD_{i,j}^{(LR)}$ denote the corresponding values of the partial dependence at point $u_{i,j}$ for RF and LR, respectively. Our ad-hoc measure is based on the absolute difference $|PD_{i,j}^{(RF)} - PD_{i,j}^{(LR)}|$ between these two quantities. To give more importance to ranges of X_j with many observations, these differences are weighted by the proportion $W_{i,j}$ of observations of feature X_j that are closer to point $u_{i,j}$ than to any other point (note that $\sum_{i=1}^{10} W_{i,j} = 1$).

Finally, to obtain a measure of the difference of partial dependence plots over the p features, each feature is weighted by its relative importance R_j in order to give more weight to informative features. The relative importance R_j is defined as the variable importance of feature X_j (or 0 if this variable importance is negative) divided by the sum of the variable importances of all features.

Our simple measure of the differences between partial dependences for RF and LR for a dataset of interest is thus defined as

$$\Delta PartialDependence = \frac{1}{p} \sum_{j=1}^{p} R_j \sum_{i=1}^{10} W_i \cdot |PD_{i,j}^{(RF)} - PD_{i,j}^{(LR)}|. \quad (1)$$

Difference in accuracies vs. difference in partial dependences for the 243 datasets

When displaying the scatterplot of Δacc vs. $\Delta PartialDependence$ for the 243 datasets included in our study, no clear trend can be identified. We subsequently select three “extreme” cases from OpenML and inspect them more closely.

As a first extreme case (Case 1), we select a dataset with low $|\Delta acc|$ and high $\Delta PartialDifference$. The second extreme case (Case 2) shows both low $|\Delta acc|$ and low $\Delta PartialDifference$. The third extreme case (Case 3) shows a very high Δacc and a high $\Delta PartialDifference$. These three datasets are investigated in detail below.

Extreme case 1: Low $|\Delta acc|$ and high $\Delta PartialDependence$

**OpenML dataset ID**	**1479**
n	1200
p	100
$accRF$	0.57
$accLR$	0.58
Δacc	0.01
$\Delta PartialDependence$	0.37
R_j (best feature)	1.8 %
R_j (2nd best feature)	1.8 %

In this case, p is large and no feature has a relative importance exceeding 1.8%. It seems that the dataset does not have enough useful information for classification, hence the relatively poor accuracies with both RF and LR. It can be seen from Figure 1 (top-right panel) that the two main features are highly correlated and insufficient to separate the two classes (depicted as blue and red points, respectively). LR does not converge and yields incoherent partial dependence patterns. RF seems to be more robust to this lack of information and to better extract information from the two best features, which is however insufficient in improving accuracy, hence the similar accuracies of RF and LR.

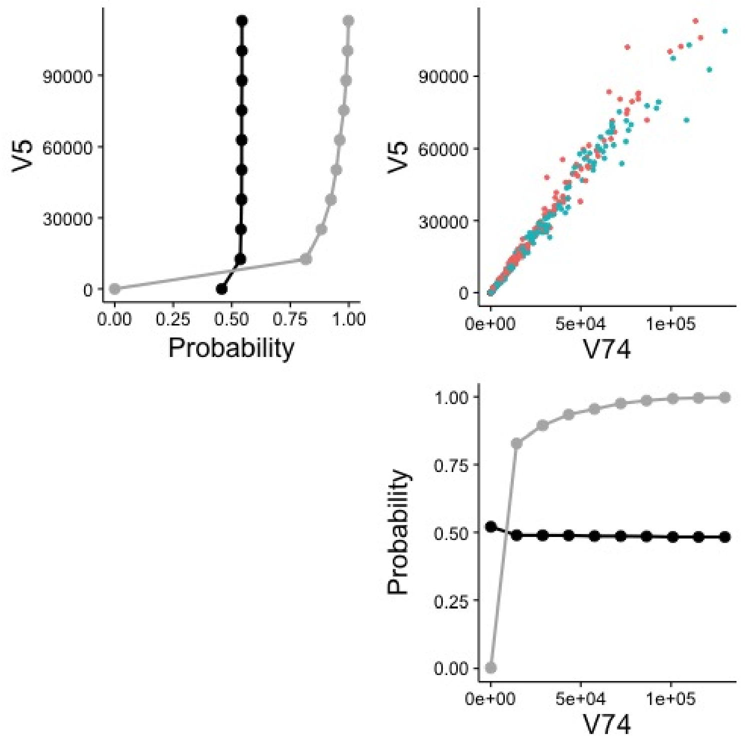

Figure 1 Partial Dependence representation for dataset ID 1479. The **top-right** panel shows the projection of the dataset on the two main features, here 'V74' and 'V5'. Red points represent the positive class and blue points the negative class. The **top-left** and **bottom-right** panels show the partial dependence plots for each of these two main features (grey = RF, black = LR).

Extreme case 2: Low $|\Delta acc|$ and low $\Delta PartialDependence$

OpenML dataset ID	923
n	10000
p	4
$accRF$	0.99
$accLR$	0.99
Δacc	0
$\Delta PartialDependence$	0.0036
R_j (best feature)	63.9 %
R_j (2nd best feature)	33.9 %

In this case the two models are very close. This is due to the linearity of the problem, as can be seen from Figure 2 (top-right panel). In this easy scenario, both algorithms perform equally well, close to perfect classification. It can be seen from Figure 2 (top-left and bottom-right panels) that RF and LR partial dependences are nearly indistinguishable for the two main features 'northing' and 'isns'.

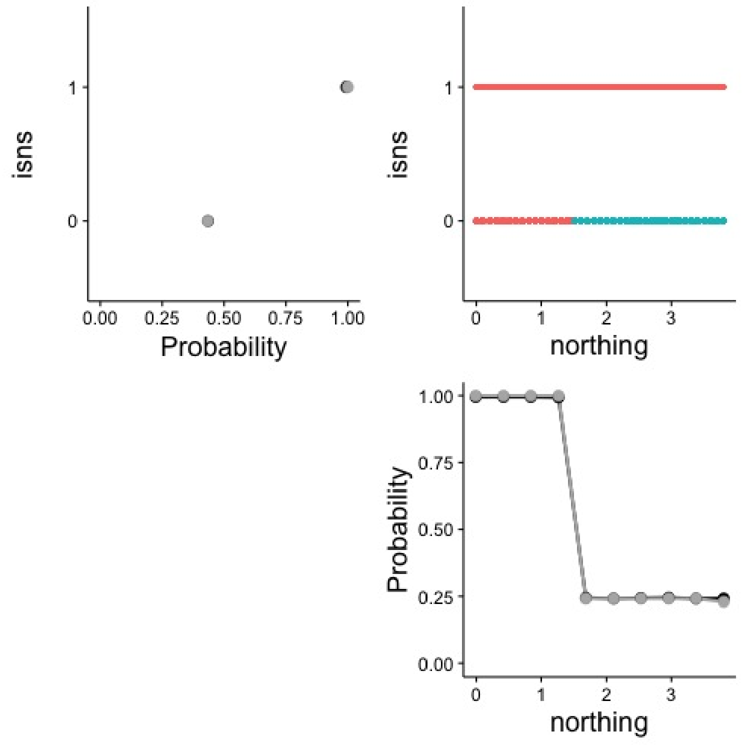

Figure 2 Partial Dependence representation for dataset ID 923. The **top-right** panel shows the projection of the dataset on the two main features, here 'isns' and 'northing'. Red points represent the positive class and blue points the negative class. The **top-left** and **bottom-right** panels show the partial dependence plots for each of these two main features (grey = RF, black = LR).

Extreme case 3 : High Δacc and high $\Delta PartialDependence$

OpenML dataset ID	1460
n	5000
p	2
$accRF$	0.89
$accLR$	0.56
Δacc	0.33
$\Delta PartialDependence$	0.13
R_j (best feature)	57.2 %
R_j (2nd best feature)	42.8 %

In this case, $p = 2$ so that we can visualize the whole dataset as 2D representation in Figure 3 (top-right panel). Δacc is large, i.e. RF performs substantially better than LR. We can clearly see a dependency in Figure 3 that explains the better performance of RF. This dependency can also be seen in the difference between partial dependences of RF and LR, especially for feature V2. This extreme case illustrates the better behaviour of RF in case of non-linear dependency structures (as also previously outlined through our simple simulation in Section 2.3).

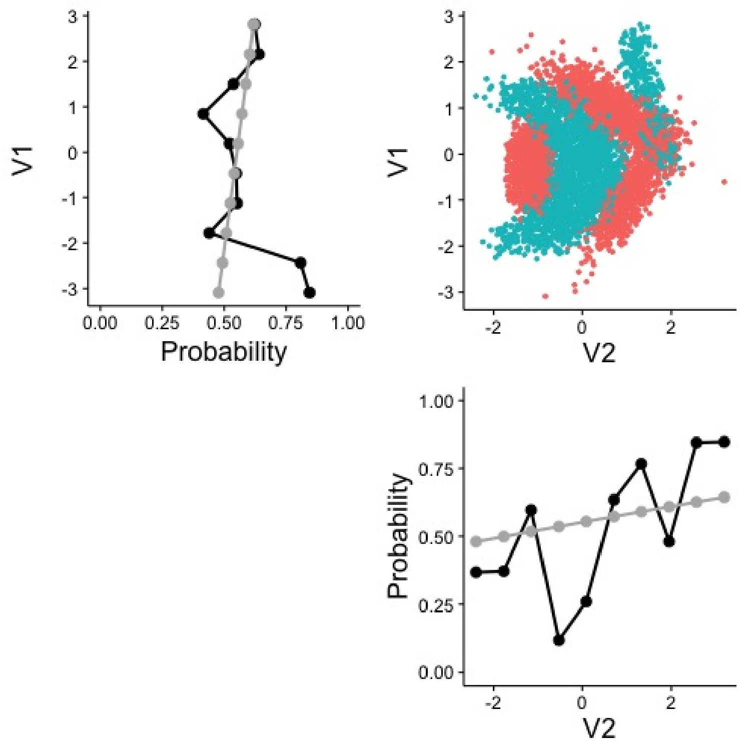

Figure 3 Partial Dependence representation for dataset ID 1460. The **top-right** panel shows the projection of the dataset on the two main features, here 'V1' and 'V2'. Red points represent the positive class and blue points the negative class. The **top-left** and **bottom-right** panels show the partial dependence plots for each of these two main features (grey = RF, black = LR).
