# Supplementary material for: Random forest versus logistic regression: a large-scale benchmark experiment
Source: BMC Bioinformatics. 2018 Jul 17;19:270. doi: 10.1186/s12859-018-2264-5 (PMC6050737; doi:10.1186/s12859-018-2264-5)
Supplement: Supplementary file 4 — Results with tuned random forest (TRF). Additional file 4 shows the results of the comparison study between LR, RF and TRF based on the 67 datasets from biosciences/medicine. (PDF 224 kb) [file 12859_2018_2264_MOESM4_ESM.pdf]

Additional file 4:
Results with tuned random forest (TRF)

Benchmark study comparing LR, RF and TRF

This figure displays in the top panel the boxplots of acc, auc and brier score of the three methods LR, RF and TRF for the 76 datasets from biosciences/medicine. Furthermore, it also shows in the bottom panel the differences Δacc, Δauc and $\Delta brier$ between RF and LR (grey) and between TRF and LR (white), respectively.

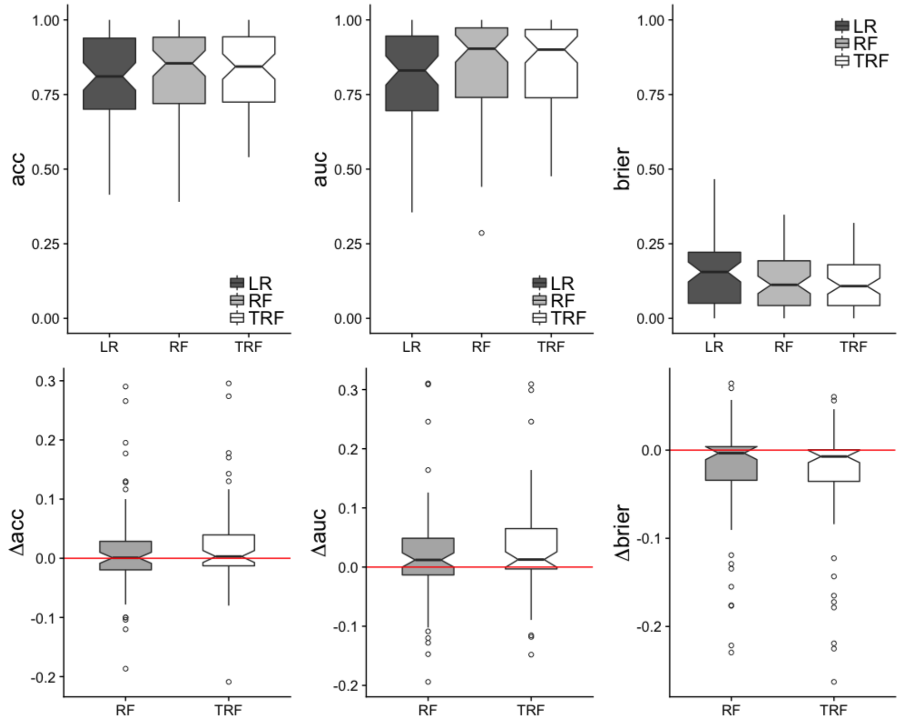

Figure 1 Main results of the benchmark experiment Boxplots of the performance for the three considered measures on the 74 considered datasets. Top: boxplot of the performance of LR (dark), RF (grey), and TR (white) for each performance measure. Bottom: boxplot of the difference of performances $\Delta perf = perf_{RF} - perf_{LR}$ (grey) and $\Delta perf = perf_{TR} - perf_{LR}$ (white).

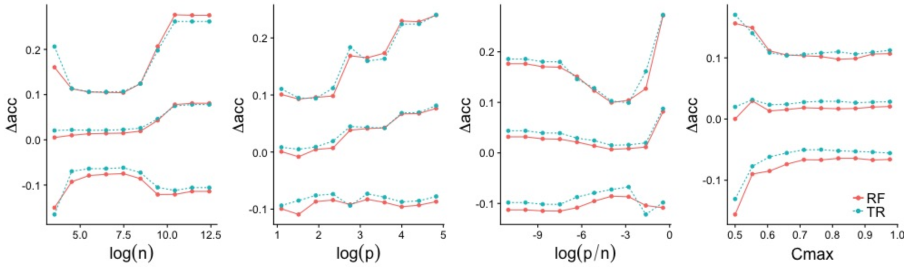

Figure 2 Partial dependence plots for the 4 considered meta-features : $log_(n)$, $log_(p)$, $log_(\frac{p}{n})$, C_{max}. The log scale was chosen for 3 of the 4 features to obtain more uniform distribution. For each plot, the middle line denotes the median of the individual partial dependances, and the lower and upper curves regions represent respectively the 25%- und 75%-quantiles.
$\Delta perf = perf_{RF} - perf_{LR}$ (red) and $\Delta perf = perf_{TR} - perf_{LR}$ (blue).

Table 1 Performances of LR and RF (for the 67 datasets from biosciences/medicine) (top: accuracy, middle: AUC, bottom: Brier score): mean performance μ, standard deviation σ and confidence interval for the mean (estimated via the bootstrap BCa method). It can be seen from this table that RF performs significantly better than LR for all three measures.

**Accuracy**	μ	σ	BCa confidence interval
Logistic regression	0.80408	0.13942	[0.76939, 0.83782]
Random forest	0.81853	0.15133	[0.78037, 0.85226]
Tune Ranger	0.82744	0.14123	[0.79109, 0.85919]
Difference RF-LR	0.01444	0.07919	[-0.00215, 0.03531]
Difference TR-LR	0.02336	0.07576	[0.00707, 0.04504]
**auc**			
Logistic regression	0.80827	0.15360	[0.76742, 0.84302]
Random forest	0.83074	0.17393	[0.78509, 0.86931]
Tune Ranger	0.83930	0.15742	[0.79609, 0.87179]
Difference RF-LR	0.02247	0.08918	[0.00342, 0.04551]
Difference TR-LR	0.03103	0.08094	[0.01359, 0.05542]
**Brier Score**			
Logistic regression	0.14910	0.10586	[0.12474, 0.17646]
Random forest	0.12479	0.09302	[0.10331, 0.14734]
Tune Ranger	0.11632	0.08599	[0.09646, 0.13707]
Difference RF-LR	-0.02431	0.06295	[-0.04033, -0.01107]
Difference TR-LR	-0.03277	0.06510	[-0.05049, -0.01936]
